# American Medical Association

**Published:** 1858-05

**Authors:** 


AMERICAN MEDICAL ASSOCIATION.—The next meeting of this body will take place at Washington City on the first Tuesday in May. A larger attendance than usual is expected.

